# Three-Dimensional (3D) Laser-Induced Graphene: Structure, Properties, and Application to Chemical Sensing

**DOI:** 10.1021/acsami.1c05614

**Published:** 2021-06-24

**Authors:** Federico Maria Vivaldi, Alexander Dallinger, Andrea Bonini, Noemi Poma, Lorenzo Sembranti, Denise Biagini, Pietro Salvo, Francesco Greco, Fabio Di Francesco

**Affiliations:** † Department of Chemistry and Industrial Chemistry, University of Pisa, via Giuseppe Moruzzi 13, 56124 Pisa, Italy; ‡ Institute of Clinical Physiology, National Research Council, via Giuseppe Moruzzi 1, 56124 Pisa, Italy; § Institute of Solid State Physics, NAWI Graz, Graz University of Technology, Petersgasse 16, 8010 Graz, Austria

**Keywords:** laser-induced graphene, graphene, biosensors, chemical sensors, glucose

## Abstract

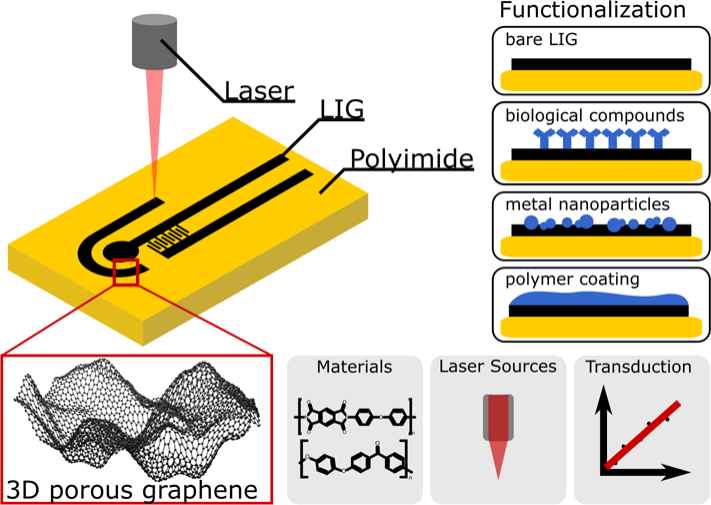

Notwithstanding its relatively recent discovery, graphene has gone through many evolution steps and inspired a multitude of applications in many fields, from electronics to life science. The recent advancements in graphene production and patterning, and the inclusion of two-dimensional (2D) graphenic materials in three-dimensional (3D) superstructures, further extended the number of potential applications. In this Review, we focus on laser-induced graphene (LIG), an intriguing 3D porous graphenic material produced by direct laser scribing of carbonaceous precursors, and on its applications in chemical sensors and biosensors. LIG can be shaped in different 3D forms with a high surface-to-volume ratio, which is a valuable characteristic for sensors that typically rely on phenomena occurring at surfaces and interfaces. Herein, an overview of LIG, including synthesis from various precursors, structure, and characteristic properties, is first provided. The discussion focuses especially on transport and surface properties, and on how these can be controlled by tuning the laser processing. Progresses and trends in LIG-based chemical sensors are then reviewed, discussing the various transduction mechanisms and different LIG functionalization procedures for chemical sensing. A comparative evaluation of sensors performance is then provided. Finally, sensors for glucose detection are reviewed in more detail, since they represent the vast majority of LIG-based chemical sensors.

## 1 Introduction

Graphene is a carbon allotrope that, since its discovery in 2004, has aroused rapidly growing interest for possible applications in many fields.^1^ It consists of a single sheet of carbon atoms arranged in a two-dimensional (2D) hexagonal lattice, where each atom shares with neighbors three in-plane σ-bonds and an out-of-plane π-bond (average interatomic distance = 1.42 Å).^2^ This peculiar structure provides graphene with outstanding flexibility, transparency, mechanical strength (42 N/m),^3^ electrical conductivity (∼1.0 × 10^8^ S m^–1^), melting point (4510 K), thermal conductivity (2000–4000 W m^–1^ K^–1^), current density (∼1.6 × 10^9^A cm^–2^), and electron mobility (200 000 cm^2^ V^–1^ s^–1^ at an electron density of ∼2 × 10^11^ cm^–2^).^4^ However, specific values are strictly dependent on the fabrication method, which could introduce structural defects or impurities.^5^ Newly minted applications of graphene sprout daily: electrical properties make it particularly suitable for electronic components, so that graphene-based sensors, capacitors, and batteries have been proposed.^6−13^ As a semimetal, graphene lacks an energy band gap, but new materials such as graphene nanoribbons overcome such limitation and pave the way even to the fabrication of digital logic devices.^14^

Graphene can be produced using techniques such as mechanical exfoliation of graphite,^15^ chemical vapor deposition onto metals from gaseous hydrocarbon precursors,^16^ and thermal decomposition and reduction of graphene oxide.^17^ Recently (Lin et al. in 2014),^18^ laser scribing, i.e,. the laser irradiation of a polymeric precursor to induce a photochemical and thermal conversion into graphene, was added to the fabrication procedures; graphene obtained in this way is usually called laser-induced graphene (LIG) or laser-scribed graphene (LSG). Yoon et al.^19^ claimed that LIG has slightly worse qualities than graphene obtained with other techniques, but relative simplicity and low cost are main advantages of laser induction. However, chemical and biosensing applications usually require further functionalization of LIG materials to enhance sensitivity and selectivity.

LIG surface morphology is characterized by a complex and inhomogeneous porous pattern resulting from the rapid generation of gases during irradiation and sp^3^ → sp^2^ conversion of carbon, typical of the planar graphene structure. The inhomogeneous structure is also due to defects in the ordered arrangement of C atoms, as 5- or 7-membered carbon rings,^18^ can be found that slightly bend the ordinary hexagonal lattice.

The composition and morphology of LIG, and, thus, its electrical, mechanical, and surface properties, are dependent on factors such as the type of laser and fabrication parameters. Recent publications from Dallinger et al.^20^ and Duy et al.^21^ described a surface obtained by a fine-tuning of laser parameters during scribing where graphene was organized in porous structures and nanofibers.

LIG has the typically high conductivity of graphene,^22^ large surface area,^23^ and resistance to strain^24^ and corrosion,^25^ but it can also be functionalized to catalyze reactions^26^ or sense pressure,^27^ temperature,^28^ intensity of magnetic field,^29^ and concentration of chemicals.

**Figure 1:**
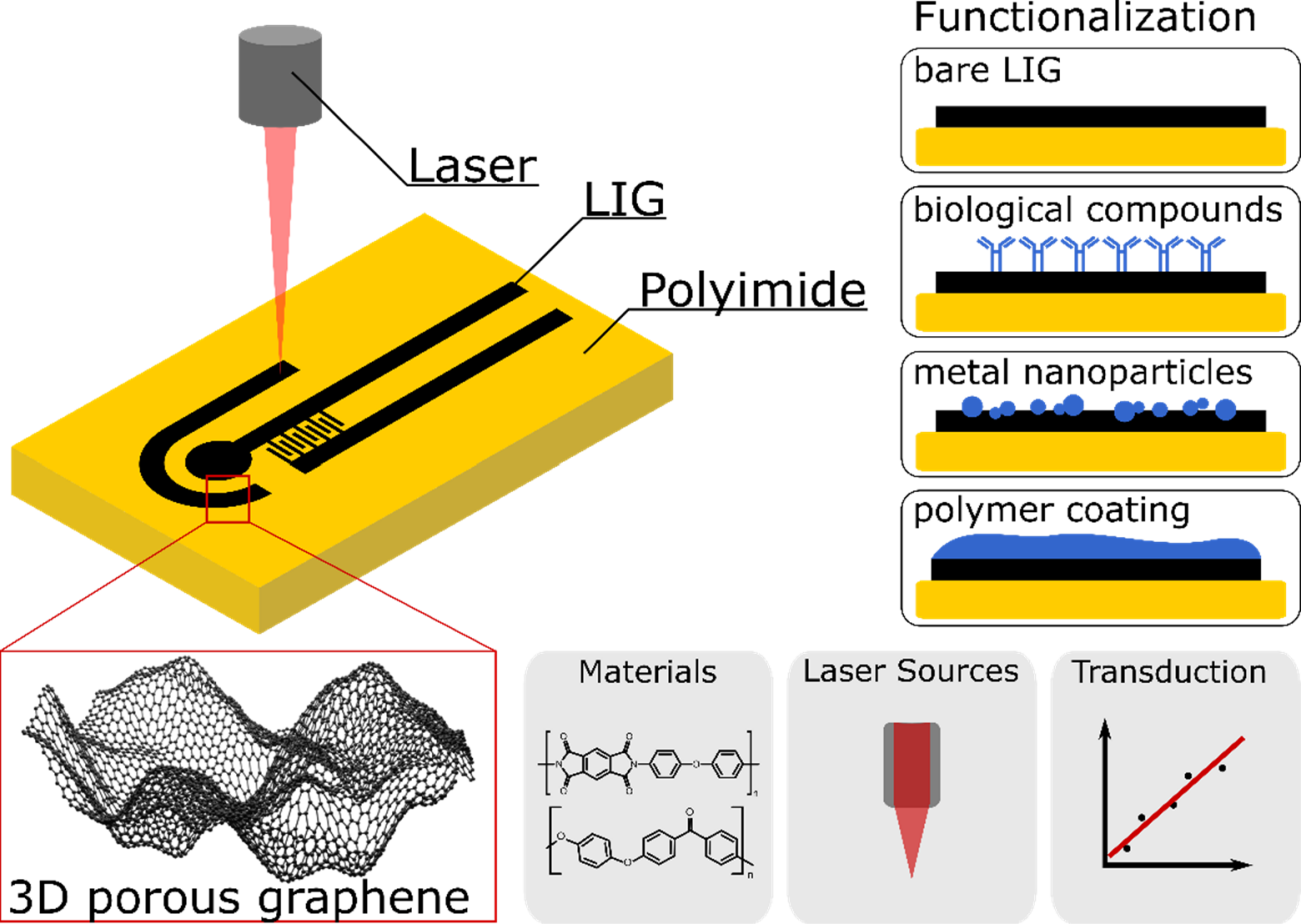
Laser-scribing of LIG on polyimide and classification of functionalization procedures for LIG-based chemical sensors.

In this work, LIG fabrication from various polymer precursors, the resulting structures, and properties are first described with a particular emphasis on transport and surface properties, then the application of these materials to the fabrication of chemical sensors and biosensors is reviewed. Chemical sensors are classified based on surface functionalization procedures (Figure 1), and sensor performances are compared whenever possible.

## 2 LIG Fabrication, Structure, and Properties

Laser scribing, i.e., the irradiation with laser pulses, transforms a suitable polymeric substrate into LIG thanks to a photothermal pyrolysis process.^18^ IR laser sources (emission wavelength ≈ 10 μm) from commercial laser cutting/engraving systems were used at first to this purpose, then the recent extension to vis and UV laser sources^30−40^ have opened new possibilities, as discussed later. Differently from traditional synthetic routes based on the pyrolysis of resins at high temperature (∼1200 °C) in an inert atmosphere,^41^ laser scribing allows the fabrication of porous carbon structures by simultaneously performing synthesis, embedding into a polymer substrate, and patterning in the form of electrodes and circuits with a fully customizable shape.

Commercial IR laser scribing systems achieve spatial resolutions of the focused beam in the tens and hundreds of micrometers, which can be reduced to a few micrometers by the vis or UV lasers used for the fabrication of miniaturized systems.^5^ This is achieved by a significantly smaller laser spot size limited by the Abbe diffraction limit and therefore smaller LIG feature size, with respect to IR. Furthermore, differences in the laser-induced pyrolysis mechanism exist: the UV laser initiates a conversion that breaks the chemical bonds directly compared to the IR lasers, which induce very high local temperatures and therefore broadens the LIG feature.^32^ This is particularly important in electrochemical sensing, where the precise patterning of miniaturized working, counter, and reference electrodes is desired.^19^ The need of minimal infrastructure (computer-controlled laser rastering system operating in air), the low cost, and the high throughput make this technology attractive for sensor fabrication and scalable to mass production.

LIG can be obtained from a variety of substrates, ranging from synthetic thermosetting and thermoplastic polymers,^18^ phenolic resins,^31^ biopolymers like lignin^42^ and even textiles, wood, or food (e.g., potato skin and bread); however, in some cases, an inert environment is needed.^43^ An even larger variety of carbon-containing materials can be converted to LIG by a multiple lasering strategy.^43^ In this case, the LIG precursor is first converted to amorphous carbon by a first laser scribing, then the amorphous carbon turns to crystalline graphene by further scribing with different fluence settings. Another approach is the use of UV lasers, which enable the conversion of even more precursors like PDMS.^34^ However, these benefits come with a drawback, such as reduced sensitivity, which is related to a smaller surface area.^44^

Polyimide (PI) films, in the form of sheets, rolls, and even adhesive tapes with a wide range of thicknesses, are readily available on the market and represent the first and most popular precursor substrates for LIG manufacturing. PI is also widely employed to produce flexible electronics, thanks to its robustness and high chemical and thermal stability,^18^ which are the very same reasons that led most investigators to choose this precursor when trying to understand the mechanism of formation, the structure, and the properties of LIG. In addition, the chemical structure of PI seems particularly suitable for the generation of LIG, because of the presence of aromatic sp^2^ carbons that are more prone to form the hexagonal graphene structure than other precursors, which just generate amorphous carbon.^45^

**Figure 2:**
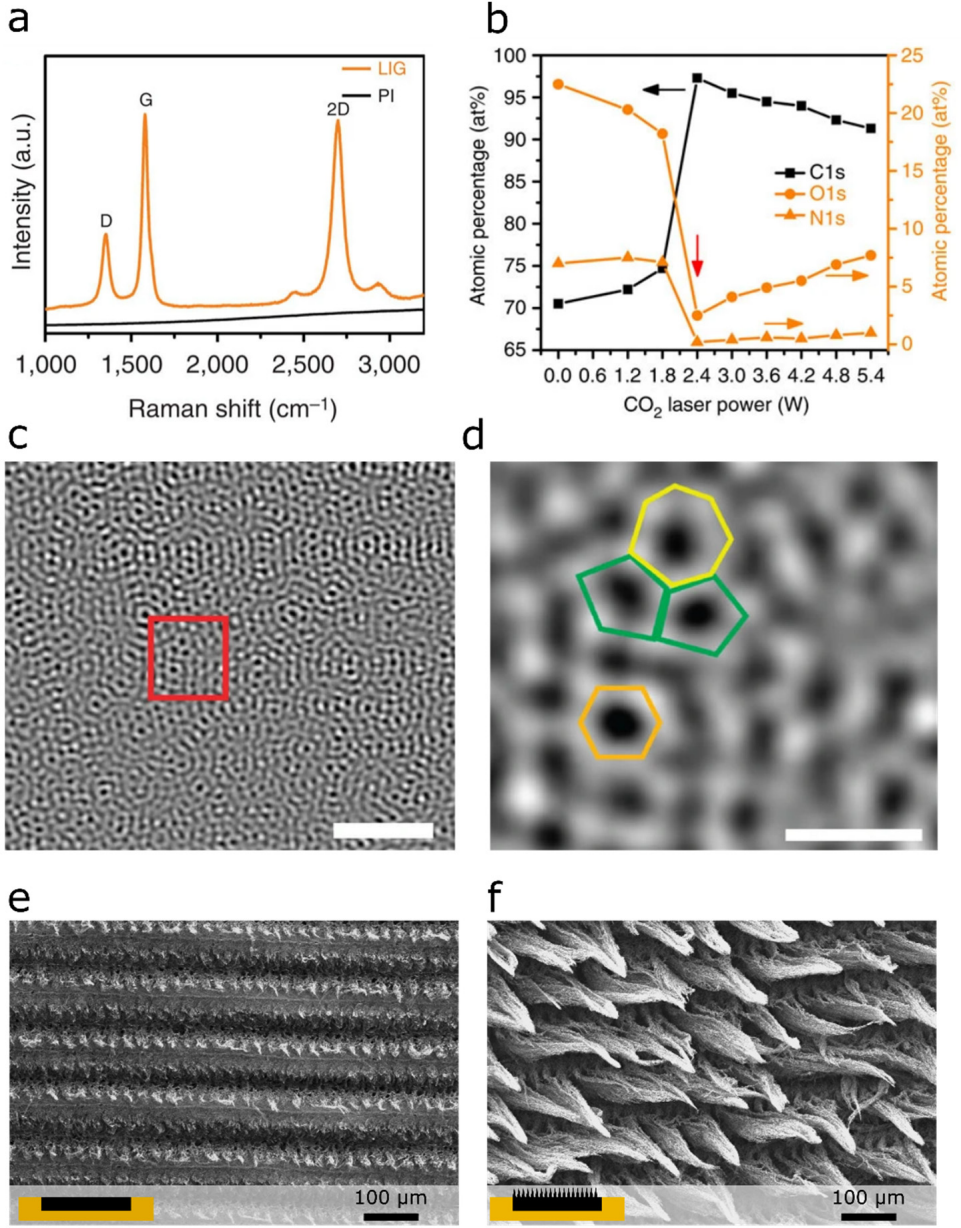
(a) Raman spectrum of LIG showing the transformation of PI to LIG; (b) X-ray photoelectron spectroscopy (XPS) of LIG scribed with different laser power showing the conversion of PI into LIG; (c, d) TEM images showing 5-, 6-, and 7-membered carbon rings; (e) SEM image of porous LIG; (f) SEM image of fibrous LIG. [Panels (a), (b), (c), and (d) have been adapted by permission from ref (18). Copyright 2014, Springer Nature. Panels (e) and (f) have been adapted from ref (20). Copyright 2020, American Chemical Society, Washington, DC.]

Raman spectroscopy of LIG formed from PI (Figure 2a) shows the typical 2D band at 2700 cm^–1^ associated with randomly stacked graphene layers, whereas the D/G band intensity ratio speaks for a high degree of carbonization.^18^

The previous assumption is confirmed by the good performance as LIG precursors of other aromatic-rich polymers, such as polyether ether ketone (PEEK),^46^ poly(ether imide) (PEI),^47,48^ various polysulfones (PSU),^49,50^ and lignin.^36,42,43,51,52^ The PI structure determines a series of properties shared with other precursors, such as a strong absorption of light in the IR and UV range, a necessary feature to enable photothermal pyrolysis, a high thermal stability and flame retardancy, which are needed to sustain the localized laser heating.

The exact mechanism of PI conversion into graphene is still unclear, despite many experimental and theoretical studies investigating laser ablation with various sources. According to Dreyfus et al.,^53^ the irradiation of polyimide with UV excimer laser pulses induces a localized heating in the target point, raising the surface temperature to ∼1700 °C when the laser fluence is 100–500 mJ/cm^2^, as determined by high-resolution laser-induced fluorescence measurements. These results are further supported by molecular dynamics (MD) simulations, suggesting that the conversion to LIG happens at high pressure and temperature.^54^ The available energy is sufficient to dissociate the C–N, C–O, C–H, and C=O bonds, as shown by X-ray photoelectron spectroscopy (XPS) (Figure 2b), and causes the release of gas and the rearrangement of aromatic fragments into a graphenic structure. Local explosions occur that determine the highly porous or fibrous structures observed. Because of the short time scale of conversion, carbon rings cannot rearrange into a perfectly regular 2D lattice structure so that 5-, 6-, and 7-membered carbon rings are produced (Figures 2c and 2d).^35,55^ LIG morphology, crystallinity, and composition are dependent on both substrate and type of laser,^56^ as well as on the actual laser fluence.^18^

The use of polymer precursors containing heteroatoms in their backbone (e.g., sulfur or fluorine), or mixed with other compounds, permits one to obtain doped LIGs and further expands the surface chemistry and the possible applications, especially in electrocatalysis and sensing fields. For example, sulfur-doped LIGs were obtained from various polysulfones (PSU) and used to fabricate membranes for the production of H_2_O_2_ or featuring antimicrobial activity;^49^ a fluorine-doped LIG was obtained from polytetrafluoroethylene (PTFE or Teflon) with a defocused lasering under Ar,^57^ a boron-doped LIG from polymer precursors mixed with boric acid,^58^ and a hybrid LIG decorated with metal oxide nanocrystals (e.g., Co_3_O_4_, MoO_2_) from polymer precursors mixed with metal complexes.^59^

Furthermore, the addition of metal salts to polymer precursors such as various phenolic resins, not prone to produce LIG in the pristine state, permitted us to obtain 3D porous LIG with good conductivity. This counterintuitive result was ascribed to metal salts such as FeCl_3_ that, on the one hand, enhanced flame retardancy and thermal stability of the polymer composite structure, and on the other had a catalytic effect in formation of graphitic structures, similar to what happens with other carbon allotropes and nanomaterials.^60^

Commercial CO_2_ lasers are often used in the fabrication of LIG because, despite low cost, and easy use and maintenance, they are quite efficient in carbonizing raw materials. In addition, the operational wavelength of CO_2_ falls in the medium- and far-IR region of the spectrum, where most substrates have large absorptions that allow a fast and efficient carbonization.^45^

The use of other lasers (e.g., UV lasers^32,33,35−39,61^) is less common but aroused more interest in recent years. Operational standards obviously require adjustments for the different power output and quantity of radiation absorbed by substrates at other wavelengths, but tuning of intensity, pulse duration, and frequency can also influence LIG morphology and properties. By applying ultrashort laser pulses, such as femtosecond lasers, it is possible to focus the LIG conversion inside the precursor and create layers of LIG by simply changing the focus point, as demonstrated by Wang et al. for stacked supercapacitors.^62^ This novel processing strategy could open new opportunities even for chemical sensing; however, the use of ultrashort laser pulses for LIG-based chemical sensors has not been reported so far.

Han et al. showed that a proper balance between power and scanning speed is of fundamental importance:^60^ a high scanning speed with low laser power does not convert PI to graphene, while an excessive power or slow scanning speed can burn the substrate.

A suitable parameter to quantify the deposited laser energy for LIG formation is the laser fluence (*H*):
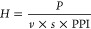
1where *P* is the power, *v* the scanning speed, *s* the spot size, and PPI the number of spots per inch. Duy et al.^21^ calculated a critical fluence of *H*_çrit_ = 5 J/cm^2^ needed for LIG formation, and by changing the laser fluence, LIG morphology can be tuned. It was shown that, for a set scribing resolution, exceeding a certain threshold fluence led to a transition from a flat, porous LIG (Figure 2e) to a species with bundles of carbon fibers up to 1 mm long and diameters of ∼100 nm emerging from the surface (Figure 2f).^20^ The same phenomenon was observed when the fluence was fixed and the scribing resolution, in terms of PPI and lines per inch (LPI), was varied. A flat LIG was obtained at high scribing resolutions, whereas similar fibers formed at low resolutions. In the former case, laser spots heavily overlap and destroy the emerging fibers, which can grow in the other case.^21^

The change in the laser fluence is also reflected in the chemical composition of the LIG. After the threshold of conversion is reached the carbon content reaches a maximum and decreases with higher fluence values (Figure 2b). At the same time, the oxygen content is increased with higher fluence values, which results in a lower-grade LIG. However, by increasing the fluence, the depth of conversion is increased and more LIG is converted; this results in an increased sheet resistance up to a certain fluence value.^18^

A surface area of 342 m^2^/g with a pore size <9 nm was measured for the flat LIG,^18^ while the surface area of the fibrous LIG was <70 m^2^/g.^21^ High-resolution images obtained by SEM showed that LIG fibers had no pores. Another important factor determining the structure and composition of LIG is the processing environment. Mamleyev et al.^63^ observed differences in LIG fabricated in air or under nitrogen atmosphere from the same PI precursor. In the former case, LIG was thicker, had a higher porosity, a slightly lower conductivity, and a hydrophilic character, whereas under N_2_ a different morphology, a higher graphitic content and a hydrophobic character were obtained. Because of these characteristics, the authors deemed the LIG prepared in nitrogen more apt for applications in electronics, because of the higher graphitic content, while LIG prepared in air was more suited in the sensor and biosensor field, because of the higher surface area. However, different results can be obtained with different precursors. Indeed, with some precursors, LIG can just be obtained when operating in an inert environment (e.g., Ar, He), as in the case of wood.^51^

While carbonizing thin PI films is ideal from the fabrication point of view, and LIG embedded in PI is suitable for several applications, it leads to problems, in terms of poor mechanical properties (cracking due to the brittleness of LIG, mismatch of elastic moduli). Thus, especially in the case of flexible/stretchable sensors, the fabrication process requires an additional step to transfer LIG onto a flexible support such as PI tape^24^ or other thermoplastic or elastomer polymer substrates.^20,64−70^ In this respect, various groups proposed different approaches.

Most flexible LIG sensors are fabricated by coating the laser-scribed PI with a liquid elastomer precursor (PDMS,^65,67,69^ Ecoflex,^68^ or PU^70^), curing the elastomer and peeling the cross-linked elastomer, together with the embedded LIG structure. This is possible because of the open porous structure of LIG: the liquid elastomer penetrates the LIG^66^ and forms a composite structure once cured. An advantage of this method is the high mechanical stability of the composite, as required in strain sensor application, but covering part of the LIG surface area with the elastomer adversely influences the performance as sensing electrodes.

Another method utilizes a sticky surface to transfer LIG onto different polymer substrates.^20,64^ The laser-scribed PI is brought in contact with the surface of a polymer carrier coated with an adhesive, and a certain pressure is applied. By changing the pressure, the amount of transferred LIG and generated damage can be controlled.^20^ Pressure ensures that all the LIG gets in contact with the glue and generates enough adhesion, then LIG is peeled off mechanically. Li et al.^71^ demonstrated a more reliable and scalable approach where laser-scribed LIG on PI was transferred and embedded onto various thermoplastic sheets in a roll-to-roll fashion by a modified thermal laminator.

Together with the intensively studied PI, phenolic resins represent another interesting precursor substrate.^31^ These materials are largely used in fields such as adhesives,^72^ constructions, material development,^73^ automotive applications, and as a precursor for preparing carbon-based materials.^74^ A large variety of thermosetting phenolic resins can be synthesized by using formaldehyde and pristine or substituted phenols; these serve as precursors for the preparation of LIG with a similar, if not slightly better, quality than LIG from PI. Indeed, a higher graphitization under the same conditions was observed.^31^

Although LIG can be produced from substrates other than PI, at present, just a few works show the use of such LIGs for chemical sensing. A particularly interesting example is the work of Lei et al.,^42^ who prepared nitrogen-doped LIG from chemically treated lignosulfonate, a byproduct of wood pulp production. The LIG obtained from such substrates displayed optimal electrical and chemical properties compared to the one made from PI. Nevertheless, it was noted that only polymers with a large quantity of lignin (>36%) lead to satisfying results.

Bioderived LIG is not only interesting in the light of environmentally friendly technology from renewable resources, but could also enable new applications in the field of disposable/degradable sensors. Especially in chemical sensing, such a platform could provide novelties in compatibility, use, and disposal in the environment and even the functionalization of living plants.

## 3 Functionalization of LIG Electrodes for Selective Analyses

Selectivity can be obtained by chemically modifying the electrode with surface elements showing strong affinity with the analyte. Because of the large surface area and presence of defects, LIG is particularly suitable for functionalization. The most popular procedures are the covalent functionalization with bioreceptors such as antibodies and enzymes, which are known for the strong and selective interaction toward target molecules, or the electrodeposition of metallic nanoparticles, which increase both surface area and conductivity of the electrode and allow stronger interactions with analytes (i.e., hydrogen on palladium nanoparticles). Electrodes can also be coated with molecularly imprinted polymers, which can selectively interact with the target analytes, thanks to specific cavities formed during polymerization on the basis of size exclusion and weak intermolecular forces. In addition, LIG surfaces doped with heteroatoms are obtained when laser induction is performed under controlled atmosphere. In most cases, this makes fabrication more complex, but selectivity of sensitive materials can be greatly enhanced. Bare LIG applications are also described in the literature, even though selectivity is poor, compared to their functionalized counterparts as discrimination is only due to the potential of the electroactive analyte, making them prone to interferents.

### 3.1 Bare LIG Electrodes as Chemical Sensors

In a few papers, bare LIG electrodes were tested as chemical sensors with good results against small and medium size analytes, mainly by voltammetric methodologies (see Table 1).

**Table 1:** Main Characteristics of Bare LIG Sensors

starting material	laser	analyte	transduction method	detection limit	dynamic range	sensitivity	ref
PI	10.6 μm	hydrazine	cyclic voltammetry	70 μM	0.1–0.5 mM	80%–115% ((Δ*I*/*I*)%)	(75)
							
PI	450 nm	trans-resveratrol	differential pulse voltammetry	0.16 μM	0.2–50 μM	0.88 μA/decade	(23)
							
PI (transfer)	10.6 μm	sulfate ions	electric impedance	–	1–10 000 ppm	0.18–0.8 W/ppm (a)	(24)
						0.035–0.065 W/ppm (b)	
PI	10.6 μm	gaseous NH_3_	chemoresistive measurement	–	75–400 ppm	0.087% ppm^–1^	(76)
							
PI	10.6 μm	uric acid and tyrosine	differential pulse voltammetry	0.74 μM^c^	3–40 μM	3.50 μA μM^–1^ cm^–2^ (c)	(77)
				3.60 μM^d^	50–500 μM^d^	0.61 μA μM^–1^ cm^–2^ (d)	
							
PI	10.6 μm	miRNA	differential pulse voltammetry	10^–9^ μM	10^–9^–10^–3^ μM	–	(78)

a Sensitivity at low frequencies at low and high concentration ranges.

b Sensitivity at high frequencies at low and high concentration ranges.

c For uric acid.

d For tyrosine.

Sharma et al.^75^ realized a simple, yet efficient and flexible, hydrazine sensor, where LIG electrodes were obtained on PI sheets and used for voltammetric analysis immediately after fabrication without any further processing step. According to the authors, the defects in the graphene structure and the resultant porosity explained the high sensitivity toward hydrazine. The sensor was also sensitive to other molecules such as glucose, formaldehyde, and ascorbic acid, although sensitivity was much lower, in comparison to hydrazine.

**Figure 3:**
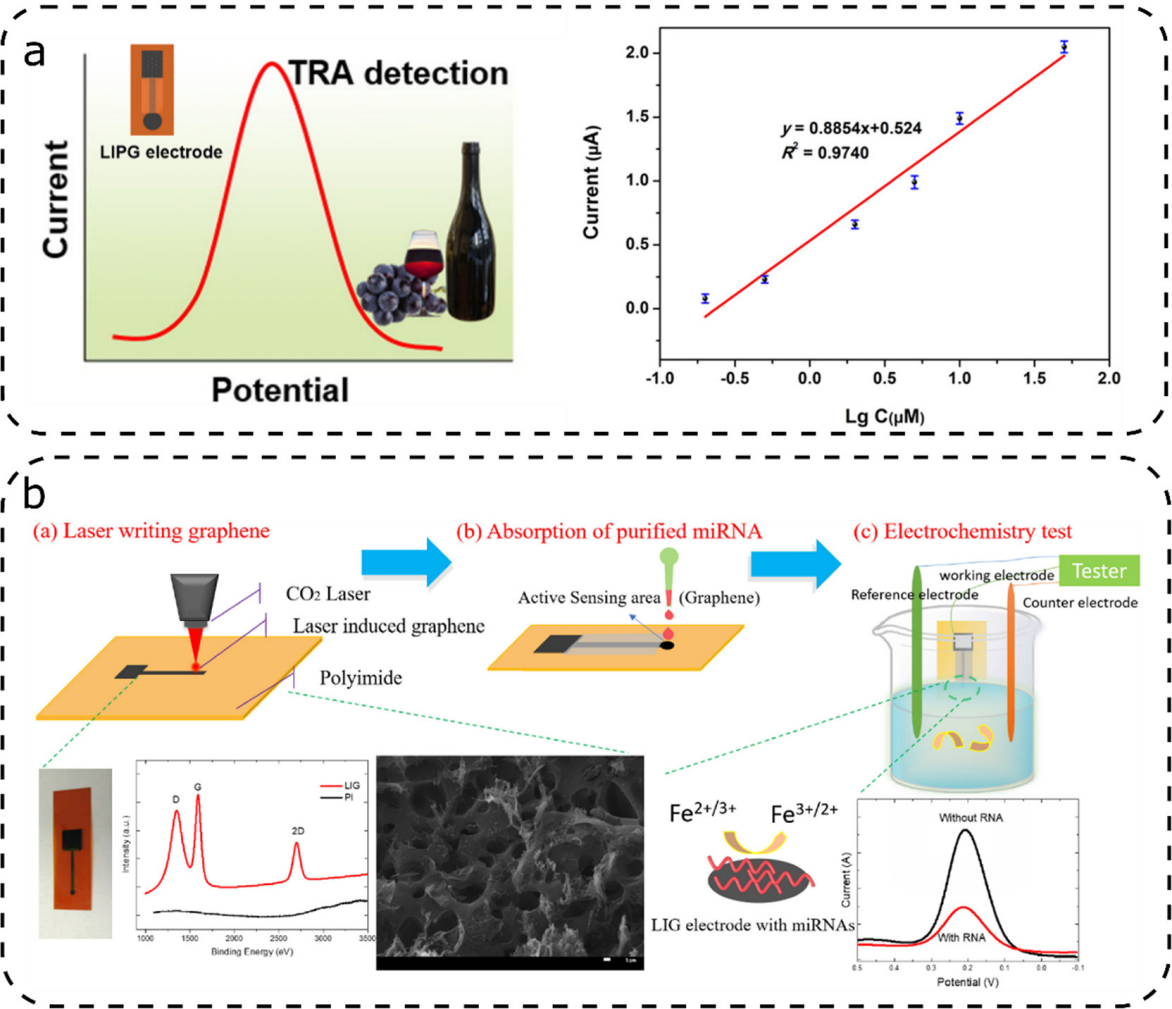
Illustrative bare LIG chemical sensors: (a) detection of trans-resveratrol, a marker of wine quality and (b) detection of micro-RNA, [Panel (a) was reprinted with permission from ref (23). Copyright 2020, Elsevier; panel (b) with reprinted with permission from ref (78). Copyright 2020, Elsevier.]

Zhang et al.^23^ developed a sensor measuring the concentration of trans-resveratrol (TRA), a marker of wine quality (Figure 3a). Untreated LIG scribed into a PI sheet was used as the sensor. A linear response to TRA concentration was obtained with this sensor using differential pulse voltammetry (DPV) and testing solutions. Tests with red wine and grape skin samples were also performed to evaluate the influence of possible interferents, in particular d-fructose, quercetin, and catechin in those matrices, but the response of the sensor to TRA did not show any matrix effect.

Han et al.^24^ proposed a method for sensing sulfate ions in water as potential application of their LIG electrodes. LIG electrodes were scribed onto a PI sheet and then transferred onto the adhesive side of a PI tape. The electrodes were dipped in solutions with different concentrations of sulfate ions in the range of 1 ppm to 10 000 ppm. Impedance measurements were performed, finding a good correlation (*R*^2^ = 0.997) between measured signal and sulfate ion concentration.

Another possible application of bare LIG on PI in sensing was reported by Wu et al.,^76^ who fabricated an ammonia gas sensor that exploited both the electrical and physical properties of the material. The resistance of a LIG track was monitored as a test gas flowed through a chamber housing the sensor, and a correlation between variation of resistance and concentration of NH_3_ in air was found. In order to enhance sensor performance, the LIG track was heated by the Joule effect to favor desorption of NH_3_ from the sensor surface and restore the initial conditions.

Yang et al.^77^ fabricated a wearable sensor to monitor the concentrations of uric acid and tyrosine in human sweat. Microfluidic channels were fabricated by laser lithography in a polyethylene terephthalate layer attached to a PI film supporting the LIG sensor that could be placed in contact with the skin for the simultaneous measurement of the sweat concentrations of the two molecules by electrocatalyzing their oxidation process.

Wan et al.^78^ developed an N-doped LIG sensor for the detection of micro-RNA (miRNA) exploiting DPV with [Fe(CN)_6_]^3–^ as a redox probe (Figure 3b). Their LIG, fabricated with a CO_2_ laser and PI, incorporated a small amount of N atoms from the PI substrate during laser induction, resulting in a slightly improved conductivity of the material and affinity to nucleic acids. Upon the dropcasting of purified miRNA onto the electrode and the performance of DPV measurements, a correlation between the concentration of miRNA and DPV signals was established.

It is clear to the reader that the nonspecific nature of LIG makes the use of bare electrodes application-oriented. In fact, to successfully perform analysis without interference, an a priori knowledge of the system under study is necessary. The presence of electroactive species different from the analyte and the effect on the signal of the latter should be known. To overcome such limitations, LIG are usually modified to improve the selectivity toward the analytical target.

### 3.2 Functionalization of LIG Electrodes with Biological Compounds To Produce Biosensors

The use of biomolecules such as enzymes, aptamers, and antibodies in chemical analysis is a prominent field of research, because of the high specificity and selectivity of such molecules. Table 2 summarizes the analytical properties of LIG-based biosensors.

**Table 2:** Main Characteristics of LIG-Based Biosensors

starting material	laser	analyte	transduction method	detection limit	dynamic range	sensitivity	ref
PI	405 nm	biogenic ammines	amperometry and cyclic voltammetry	7.70 ± 2.80 μM (a)	50 μM to 1.6 mM	58.7 ± 5.90 μA/mM (a)	(79)
				11.6 ± 2.60 μM (b)		23.3 ± 1.90 μA/mM (b)	
							
PI	10.6 μm	urea	potentiometry	10 ^–4^ M	10^–4^–10^–1^ M	–	(63)
							
PI	10.6 μm	thrombin	differential pulse voltammetry	10^–6^ μM (c)	1–100 pM	–2.4 ± 0.16 μA cm^–2^/decade (c)	(80)
				5 × 10^–6^ μM (d)		–3.9 ± 0. μA cm^–2^/decade (d)	
						–2.5 ± 0.04 μA cm^–2^/decade (e)^g^	
							
PI	10.6 μm	thrombin	electrochemical impedance spectroscopy	0.12 pM	0.01–1000 nM	–	(81)
							
PI	10.6 μm	*S. enterica Typhimurium*	electrochemical impedance spectroscopy	10 CFU mL^–1^ ( c)	25–103 CFU mL^–1^ (c)	42 Ω log CFU^–1^ ( c)	(82)
				13 ± 7 CFU mL^–1^ (f)	25–105 CFU mL^–1^ (f)	24 Ω log CFU^–1^ mL (f)	

a Sensor made with high quality materials.

b Sensor made with locally sourced materials.

c In buffer.

d In serum.

e In serum with interferents

f In chicken broth.

g The sensitivity of this sensor in both buffer and serum heavily depends on the concentration of the analyte, all the sensitivity values are per logarithmic concentration unit.

Vanegas et al.^79^ used a combination of copper microparticles and the enzyme diamine oxidase for the detection of biogenic ammines in food. The electrodeposition of metal particles is a common technique used to enhance LIG sensing properties. After laser induction, copper sulfate was used to electrodeposit copper microparticles on the LIG surface and the enzyme was successively bound to the electrode. The deamination of ammines promoted by the enzyme produces ammonia, aldehydes, and H_2_O_2_. The latter is then decomposed at the electrode surface in water and oxygen, producing a measurable current, whose intensity was correlated to the concentration of biogenic ammines (or ammines in general). In particular, the authors showed that the sensor was particularly sensitive to three ammines: histamine, putrescine, and cadaverine.

Mamleyev et al.^63^ produced a small flexible sensor capable of detecting urea, by exploiting the urease enzyme. In this case, the laser induction process was performed with a far-IR laser. A chitosan gel was electrodeposited onto the electrode to simulate a cellular matrix and preserve the activity of the enzyme, thus extending sensor life. The enzyme was then cross-linked onto the surface with glutaraldehyde. This sensor exploited the potentiometric measurement of pH: the enzyme urease catalyzed the decomposition of urea into CO_2_ and NH_3_, and the increase in pH caused by the NH_3_ was correlated to the presence of urea in the solution.

**Figure 4:**
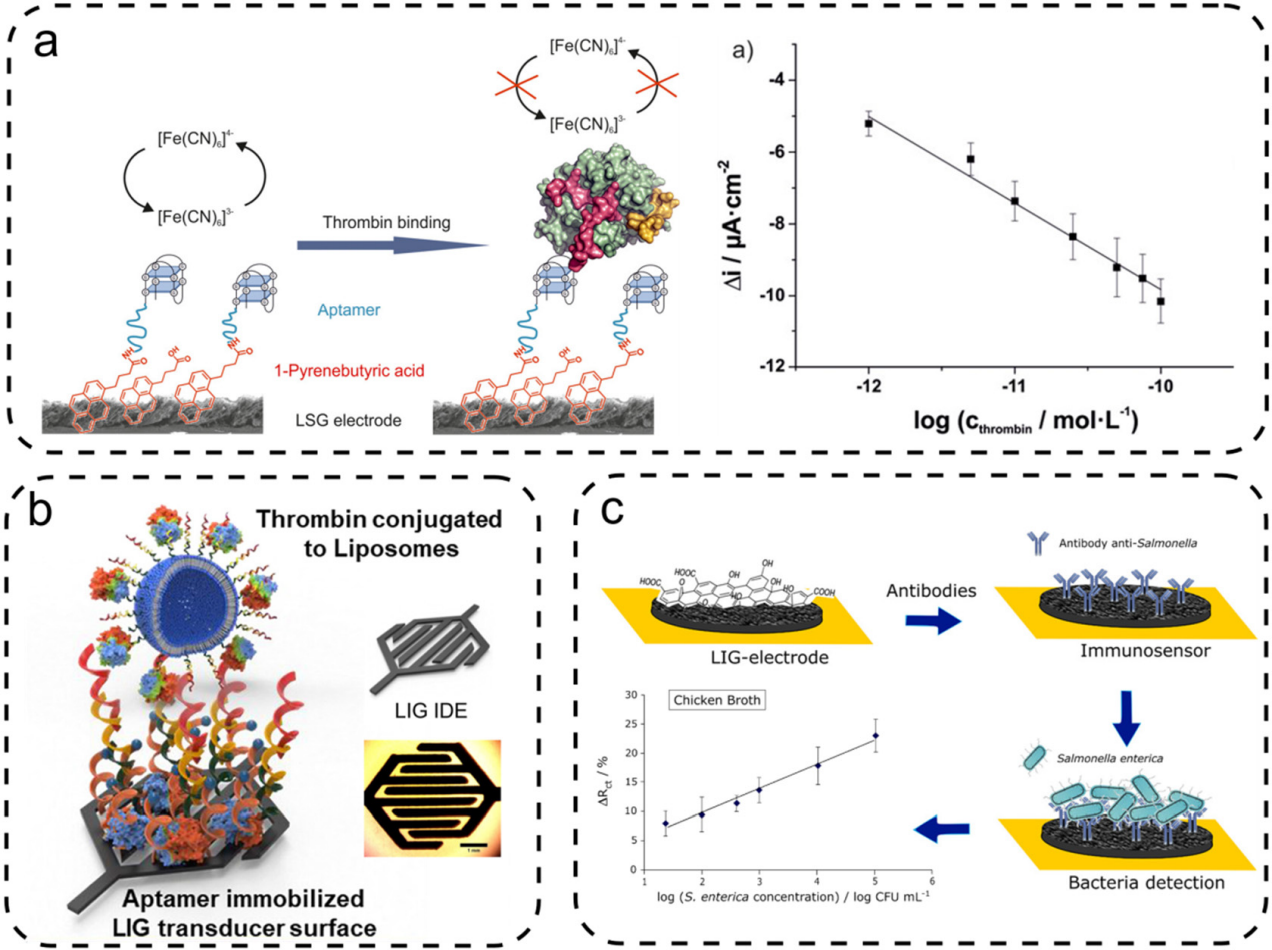
Illustrative LIG biosensors: (a) aptamer-based sensor using differential pulse voltammetry (DPV) to detect thrombin; (b) aptamer based sensor using electrochemical impedance spectroscopy, and (c) LIG immunosensor using antibodies to detect *Salmonella enterica*. [Panel (a) was reprinted from ref (80). Copyright 2020, American Chemical Society, Washington, DC. Panel (b) was adapted, with permission, from ref (81). Copyright 2020, Elsevier. Panel (c) was adapted, with permission, from ref (82). Copyright 2020, American Chemical Society, Washington, DC.]

Fenzl et al.^80^ used aptamers to detect thrombin, an enzyme promoting coagulation (Figure 4a). Their LIG electrode was prepared by laser induction of a PI foil and subsequently treated with 1-pyrenebutirric acid, which is a cross-linker used to covalently bind the aptamer. DPV was the selected transduction technique, and the peak current of ferrocyanide redox reaction was recorded before and after incubation of the sensor with the sample containing thrombin. Furthermore, the authors verified the negligible effect on the sensor of bovine serum albumin as nonspecific adhesive proteins in real samples.

Yagati et al.^81^ also used aptamers for thrombin detection. For this LIG was scribed on PI as interdigitated electrodes (Figure 4b). The concentration of thrombin was then measured by means of electrochemical impedance spectroscopy (EIS). This technique measured the change in capacitance due to the interaction of thrombin with the aptamer. It was found that the capacitive LIG sensor was faster and more sensitive than sensors using amperometric detection.

Soares et al.^82^ modified the surface of LIG scribed into PI with salmonella antibodies (Figure 4c). The LIG surface was functionalized by carbodiimide cross-linking of polyclonal antibodies. A Nyquist plot was recorded by EIS and the charge transfer resistance (R_ct_) was calculated. The change in R_ct_ was used to create a calibration curve for the concentration of bacteria. To assess the selectivity of the sensor, the signal in the presence of food pathogens (*E. coli*, *P. aeruginosa*, *B. cereus*, *S. aureus*, and *L. monocytogenes*) was recorded, showing no significant variation of the *R*_ct_ value.

### 3.3 Functionalization of LIG Electrodes by Electrodeposition of Metal Nanoparticles

Decoration of LIG by the electrodeposition of metal nanoparticles (copper, gold, platinum, and palladium) is commonly used to improve the electrical properties of the electrodes and enhance the performance of sensors. Metal nanoparticles increase the current density, because of both electrocatalytic effects and increased surface area. The possible use of these sensors reported in the literature range from monitoring of organic metabolites to the detection of inorganic compounds such as gaseous hydrogen or H_2_O_2_ (see Table 3).

**Table 3:** Summary of Characteristics and Comparison of LIG-Based Sensors Functionalized with Metal Nanoparticles

starting material	doping material	laser	analyte	transduction method	detection limit	dynamic range	sensitivity	ref
PI	Au and Pt NPs	10.6 μm	dopamine	cyclic voltammetry	75.0 nM	0.95–30 μM	865.8 μA mM^–1^ cm^–2^	(83)
								
PI	Pt NPs	10.6 μm	dopamine, ascorbic acid, and uric acid	cyclic voltammetry	6.10 μM^a^	–	250.7 μA mM^–1^ cm^–2^ (a)	(84)
					0.07 μM^b^	–	6995 μA mM^–1^ cm^–2^ (b)	
					0.22 μM^c^	–	8289 μA mM^–1^ cm^–2^ (c)	
								
PI	Pt NPs	10.6 μm	H_2_O_2_	cyclic voltammetry	0.2 μM	0.5 μM to 5.0 mM	248.4 μA mM^–1^ cm^–2^	(85)
								
PI	Pt NPs	10.6 μm	methane	cyclic voltammetry	9 ppm	1–50 ppm	0.55 μA ppm^–1^ cm^–2^	(86)
								
PI	Pd NPs	10.6 μm	gaseous H_2_	chemoresistive measurements	–	–	–	(64)

a For ascorbic acid

b For dopamine.

c For uric acid.

**Figure 5:**
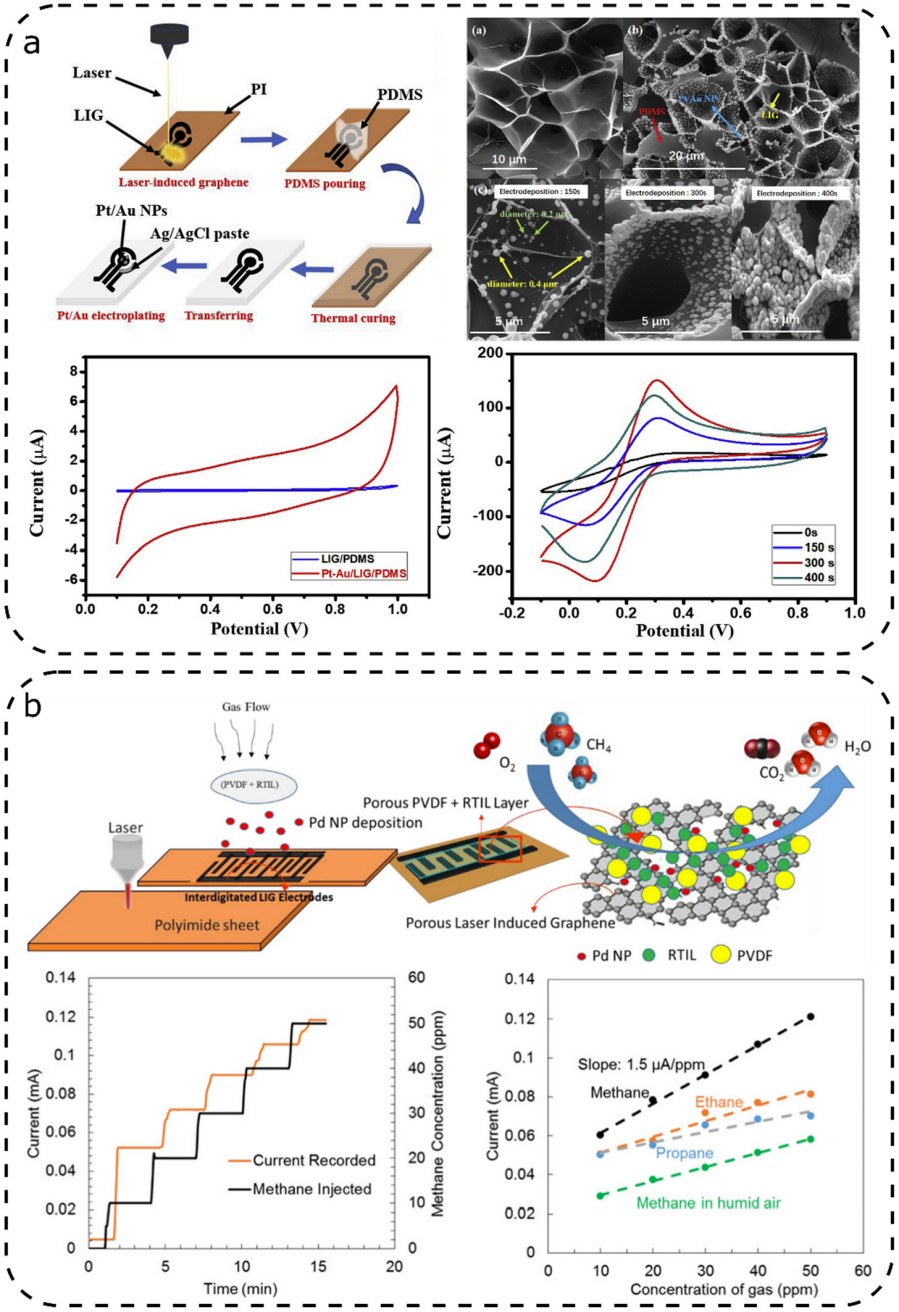
Illustrative LIG chemical sensors using metal nanoparticles: (a) sensor using Pt/Au NP to detect dopamine and (b) interdigitated LIG electrodes with Pd NP capable of detecting methane and other gases. [Panel (a) was adapted, with permission, from ref (83). Copyright 2019, Elsevier; panel (b) was reproduced, with permission, from ref (86). Copyright 2019, American Chemical Society, Washington, DC.]

Hui et al.^83^ presented a device that combined gold and platinum nanoparticles and was capable of detecting low amounts of dopamine and could differentiate from known interferents, such as uric and ascorbic acid (Figure 5a). Besides improving the conductivity of the electrode, surface nanoparticles electrocatalyzed the redox reactions during cyclic voltammetry measurements. Subsequent studies showed that the sensor was capable of correctly quantifying dopamine, despite the possible presence of interferents with similar oxidation potential. A final test performed in a complex matrix such as human urine showed that it was able to detect dopamine without being affected by interferents.

Nayak et al.^84^ reported the development of a LIG-based device for the analysis of dopamine, ascorbic acid, and uric acid. To improve the electrocatalytic effect toward the analytes, Pt nanoparticles were deposited on the carboneous surface. However, the paper is focused on the electrocatalytical performance of the device and real samples experiments were not performed.

Also, Zhang and co-workers used electrodeposited platinum on their LIG electrodes. The H_2_O_2_ concentration in a deoxygenated phosphate buffer was measured by reducing it into H_2_O and measuring the current associated with the reaction with cyclic voltammetry.^85^ Metal nanoparticles were deposited on to the LIG electrode by RF magnetron sputtering, a technique where argon ions are accelerated by an electric field against a layer of material (platinum in this case) to make it sputter onto a desired surface. Different from electrodeposition, the use of the magnetron allowed the whole sensor surface to be covered with a homogeneous Pt layer. The layer thickness affected sensor properties, and researchers concluded that the optimal thickness was ∼15 nm. Sensitivity and detection limit of this H_2_O_2_ sensor were comparable to those of other sensors in the literature, but the dynamic range was narrower.

Dosi et al.^86^ used a Pd nanoparticle solution to decorate LIG for methane detection (Figure 5b). Interdigitated electrodes were scribed in PI and, after decoration with Pd nanoparticles, the electrodes were covered by a solid polymer-electrolyte based on polyvinylidene fluoride (PVDF) with 1-ethyl-3-methylimidazolium TFSI (EMImTFSI). The recorded current, caused by the electro-oxidation of methane, was linearly dependent on methane concentration. The sensor also showed response to other gases such as ethane and propane but with a lower sensitivity. Furthermore, the sensor’s sensitivity was lowered by the presence of humidity. As a remark, the reported sensor showed a sensitivity 4 orders of magnitude higher than that of similar sensors operating at room temperature.

Exploiting a different transduction method, Zhu et al.^64^ proposed a resistive sensor combining LIG and palladium for monitoring gaseous H_2_. Hydrogen molecules are slowly absorbed into Pd, altering the resistance of the sensor. The effect of interfering gases such as NO_2_ and NH_3_ was investigated. The sensor showed no response toward those interferents. However, no additional analytical parameters were reported, making comparison with similar devices difficult.

### 3.4 Electrodes Based on LIG with Polymer Coating

Polymers can be used in combination with LIG to produce innovative composites under controlled conditions. Functionalization of LIG electrodes with polymers in the presence of the analyte, which is then washed away, increases sensor selectivity by molecular imprinting. Other methods such as electrodeposition or spray coating allow the use of polymers to prepare specific sensing surfaces for a variety of analytes (see Table 4).

**Table 4:** Summary of Characteristics and Comparison of LIG-Based Sensors Functionalized with Polymers

starting material	doping material	laser	analyte	transduction method	detection limit	dynamic range	sensitivity	ref
PI	PEDOT	10.6 μm	dopamine	cyclic voltammetry	0.33 μM	1–150 μM	0.22 ± 0.011 μA μM ^–1^	(87)
								
PI	eriochrome black T and polypyrrole	10.6 μm	amoxillicin and ascorbic acid	cyclic voltammetry and square wave voltammetry	11.98 nM (a)	50 nM to 100 μM (a)	–13.32 μA/decade (a)	(88)
						1.5–4 mM (b)	1.356 μA/decade (b)	
								
PI	poly- eriochrome black T and PEDOT	10.6 μm	chloramphenicol	cyclic voltammetry and EIS	0.62 nM	1 nM to 10 mM	–	(89)
								
PI	polypyrrole	10.6 μm	Bisphenol A	differential pulse voltammetry	8 nM	0.05–5 μM	26.79 μA/decade	(22)
								
PI (transfer)	polyaniline	10.6 μm	pH	potentiometry	-	pH 4–10	–53 mV/pH	(90)

a For amoxillicin

b For ascorbic acid.

Xu’s et al.^87^ proposed a polymer-based sensor dedicated to dopamine detection. This sensor was fabricated by modifying a LIG electrode by electrodeposition of poly(3,4-ethylenedioxythiophene) (PEDOT). Cyclic voltammetry was used as a transduction technique and the peak current relative to the oxidation of dopamine was measured. A correlation between the concentration of dopamine and the current was established. The selectivity of the sensor was investigated in the presence of ascorbic acid and uric acid. The dopamine peak proved to be well-resolved, even when such electroactive compounds were available in solution.

**Figure 6:**
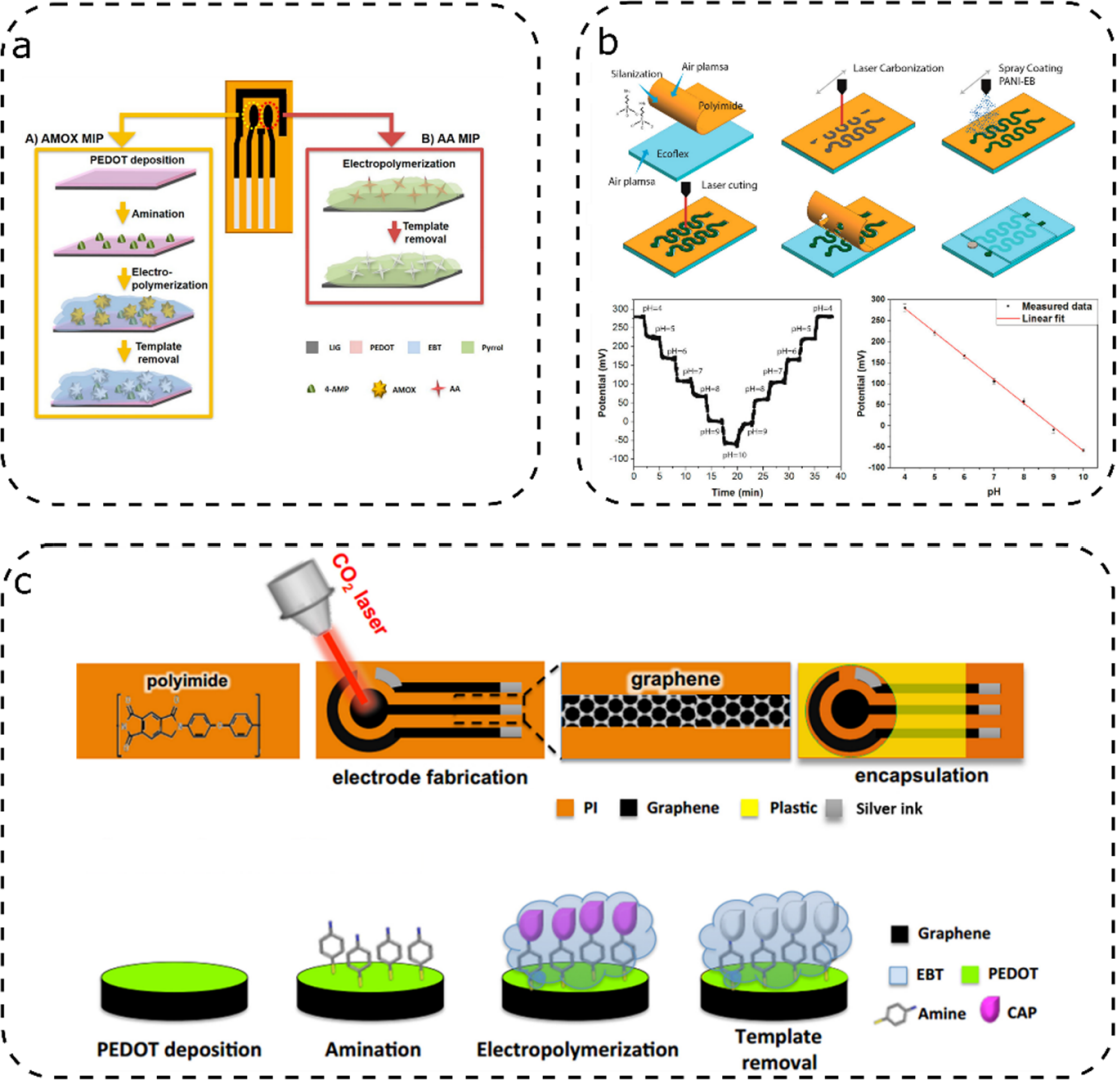
Illustrative LIG sensors with polymer coating: (a) LIG sensor fog amoxicillin and ascorbic acid using molecularly imprinted polymers,[Panel (a) was adapted with permission from ref (88). Copyright 2020, American Chemical Society, Washington, DC; adapted with permission from,^90^ Copyright 2020 American Chemical Society c) LIG electrodes using EBT for detb) flexible LIG pH sensor using polyaniline,ecting chloramphenicol, a pollutant antibiotic, adapted from,^89^ Copyright 2019, Elsevier.

Marques’s group fabricated a sensor for amoxicillin and ascorbic acid using molecularly imprinted polymers (Figure 6a).^88^ With this technique, polymerization is performed in the presence of the analyte: their interaction during polymerization and the successive washing create cavities that grant the polymer an affinity for the analyte. In their fabrication process, different procedures were used to treat LIG electrodes and make them sensitive to amoxillicin or ascorbic acid. For the amoxicillin detection, eriochrome black T was electropolymerized on the LIG electrode in the presence of the analyte. A similar process was used to add a second working electrode sensitive to ascorbic acid. Differently from the amoxicillin sensor, polypyrrole was selected as an imprintable polymer for ascorbic acid detection.

In a control experiment, the sensor was produced without the imprinting procedure. While the response of the imprinted sensor was linear and accurate, the response of the nonimprinted sensor showed no correlation. The effective sensor measured without significant errors the concentrations of amoxillicin or ascorbic acid in both artificial binary solutions and real samples.

A similar working principle was proposed by Cardoso’s group, who used poly(eriochrome black T) and PEDOT as imprinting polymers for chloramphenicol, a pollutant antibiotic (Figure 6c).^89^ LIG electrodes were previously coated with PEDOT by electropolymerizing PEDOT monomers. Subsequently, the electrode was left in a solution of 4-aminotiphenol to prepare the chloramphenicol binding sites. Eriochrome black T was electropolymerized along with chloramphenicol, which was later removed with acetonitrile. This sensor was used for cyclic voltammetry and EIS measurements. Sensor performances were compared with those of commercial screen-printed electrode sensors. The sensor fared very well against commercial sensors, showing both higher currents and narrower peaks separations; the comparison with the nonimprinted sensor highlighted the difference in quality granted from the molecular imprinting process. Furthermore, the sensor showed sensitivity toward chloramphenicol even in the presence of interferents such as sulfadiazine, amoxillicin, and oxytetracycline.

Beduk et al.^22^ developed a sensor using molecularly imprinted polypyrrole to monitor bisphenol A (BPA). In the fabrication process, the LIG electrode surface not intended for functionalization was protected by a layer of polydimethylsiloxane. Pyrrole was then electropolymerized onto the surface of the working electrode along with BPA, the procedure was finalized by dipping the electrode into a solution of acetic acid and methanol to remove BPA molecules and obtain the molecularly imprinted polypyrrole. The sensor was used for both cyclic voltammetry and differential pulse voltammetry and its performance was compared to a nonimprinted sensor. The sensing capacity of this sensor was consistent with other sensors made for the same purpose. The study on the possible interferents showed a potential fault of the sensor: the presence of interferents in fact had a noticeable effect on the measured current.

An additional work worth of mention is from Rahimi et al.,^90^ who fabricated a flexible pH sensor using polyaniline (Figure 6b). The LIG electrodes were fabricated through laser induction with a CO_2_ laser from PI laminated with another commercial polymer. A polyaniline coating was sprayed onto the electrode with a mixture of DMSO, forming a polyaniline–carbon composite. The remaining PI layer was removed, and after preparing a reference electrode with Ag/AgCl onto one of the electrodes, the sensor was ready for use. This device was designed to measure pH in biological ranges and used as a sensor directly on the skin; tests showed a linear response in the pH range of 4–10. Biocompatibility tests that showed no toxicity toward cells were performed to avoid risks of harmful effects on humans.

## 4 Glucose Detection

The main application of glucose sensors is the monitoring of glucose levels in blood or other biofluids collected from diabetic patients. These sensors should be used in harsh conditions, for example attached or below human skin and being subject to a constant strain and other stress sources and maintain its operativity over time. Cyclic voltammetry is the transduction method used in all sensors compared in Table 5, where the quantity measured is the peak current corresponding to the redox system of the electrode.

**Table 5:** Summary of Characteristics and Comparison of LIG-Based Glucose Monitoring Sensors

starting material	laser	functionalization method	transduction method	detection limit	dynamic range	sensitivity	ref
phenolic resin	405 nm, 10.6 μm^a^	ferrocene formic acid and chitosan with glucose oxidase	cyclic voltammetry	–	0.2–10 mM	–	(31)
							
PI	not specified	Cu nanoparticles	cyclic voltammetry	0.4 μM	1 μm to 6.0 mM	495 μA mM^–1^ cm^–2^	(91)
							
PI	10.6 μm	Pt nanoparticles and chitosan with glucose oxidase	cyclic voltammetry	0.3 μM	0.3 μM to 2.1 mM	65.6 μA mM^–1^ cm^–2^	(19)
							
PI	10.6 μm	Pt and Au nanoparticles, chitosan with glucose oxidase	cyclic voltammetry	5.0 μM	0–1.1 mM	6.40 μA mM^–1^ cm^–2^	(67)
							
PI	10.6 μm	Pt@Pd nanoparticles, chitosan with glucose oxidase	chronoamperometry	3 μM	3 μM to 9.2 mM	247.3 μA mM^–1^ cm^–2^	(92)
							
lignin	10.6 μm	MXene, Prussian Blue and chitosan with glucose oxidase	chronoamperometry	0.3 μM	10 μM to 5.3 mM	49.2 μA mM^–1^ cm^–2^	(42)

a Both lasers were used for the preparation of LIG in this research.

**Figure 7:**
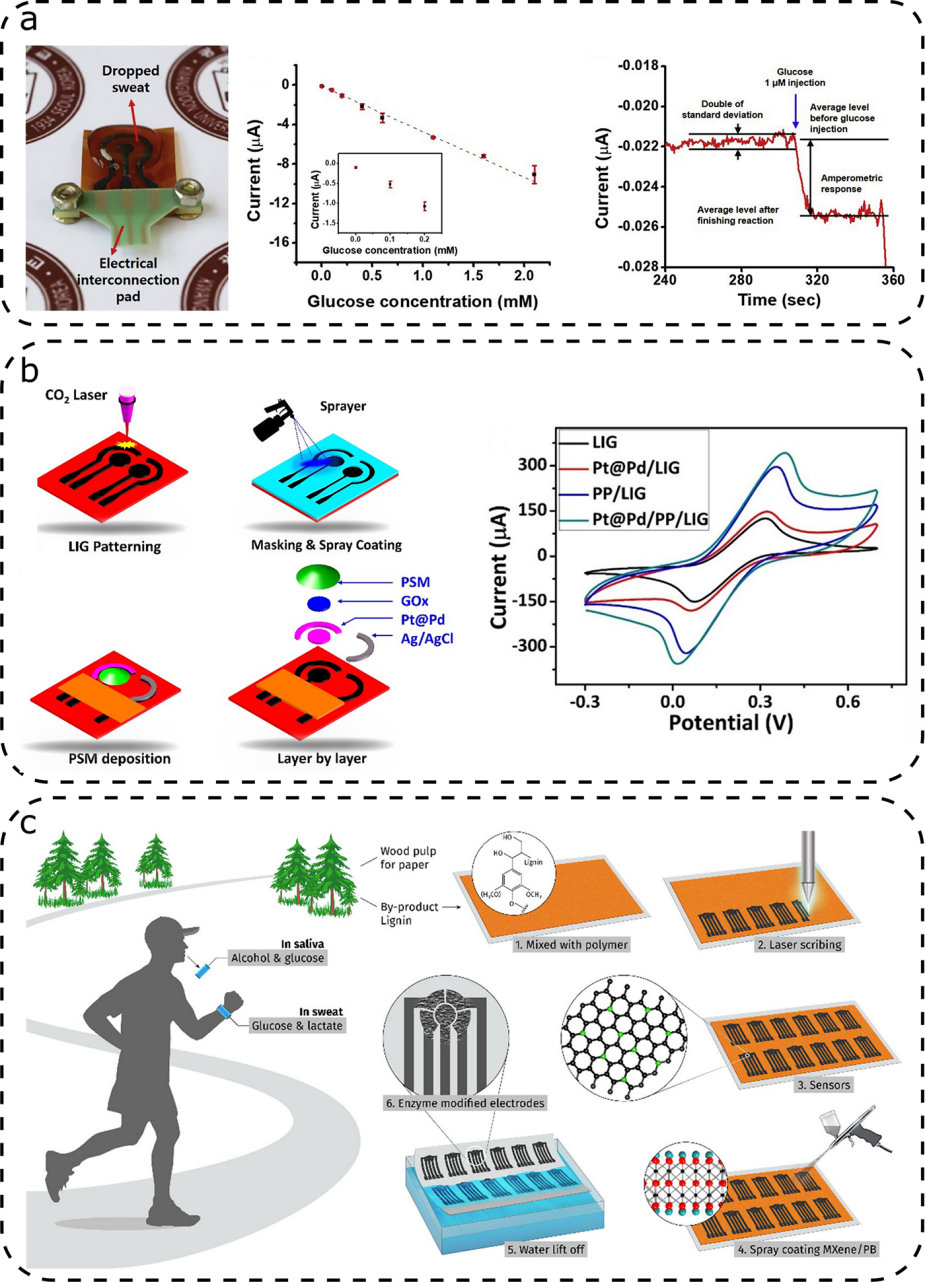
Illustrative glucose sensors based on LIG: (a) glucose detection in sweat by oxidation of glucose by glucose oxidase; (b) LIG electrodes with spray-coated PEDOT:PSS and electrodeposited Pt@Pd nanoparticles for glucose detection; and (c) lignin-based glucose sensor. [Panel (a) was adapted from ref (19). Copyright 2020, Elsevier. Panel (b) was adapted from ref (92). Copyright 2020, Elsevier. Panel (c) was adapted, with permission, from ref (42). Copyright 2020, American Chemical Society, Washington, DC.]

In most cases, the monitored redox reaction is the simple oxidation process of the H_2_O_2_ generated from the oxidation of glucose by glucose oxidase (Figure 7a).^19,31,42,67^ However, there are some exceptions, as reported in Zhang’s works.^31,91^ In the first work, copper deposited on the electrode catalyzes the direct oxidation of glucose, whereas in the second work, ferrocene formic acid was used as an electron mediator to shuttle electrons from glucose oxidase toward the electrode surface. It is interesting to note that, despite being functionalized only with copper nanoparticles and without the use of any enzyme, unlike the other four sensors, Zhang’s sensor^91^ achieved the highest sensitivity and the broadest dynamic range.

Zahed et al.^92^ reached a similar high sensitivity by spray-coating PEDOT:PSS onto the LIG and electrodepositing Pt@Pd nanoparticles (Figure 7b), followed by the deposition and immobilization of glucose oxidase and deposition of a permselective membrane layer. It was observed that the deposition of Pt@Pd nanoparticles increased the anodic and cathodic peaks in the CV curve. Although the mechanism behind this catalytic effect is not yet fully understood, it is a promising approach.

An interesting exception was demonstrated by Lei et al.,^42^ who scribed LIG electrodes for glucose detection on a film of lignin, PVA, and urea (Figure 7c). The working electrodes were modified with MXene, Prussian Blue by spray coating and chitosan with glucose oxidase. The sensitivity was in the same range as the sensors reported by Zhang.^31,91^

Compared to the recent literature about glucose sensing, the nonenzymatic LIG-based glucose sensors show similar analytical performance.^93^ Similarly, the enzymatic LIG sensors present values of sensitivity, range of lineary, and LOD close or superior to the non-LIG counterpart.^94^ These results can be explained taking into account the high surface of LIG, which grants high current densities for electrochemical sensing. These performances, combined with the simple fabrication procedure of LIG electrodes, prove the possibility of using LIG electrodes as a platform for competitive sensors.

## 5 Conclusion and Future Perspectives

Laser induction could overcome some of the problems related to the graphene production, so many researchers have focused attention on the evaluation of properties of LIG materials and their possible applications. So far, PI is considered the best raw material for LIG production, being inexpensive and having the right properties for laser induction. Yet, as mentioned by Zhang,^31^ one of the most interesting aspects of using phenolic resin instead of PI is the possibility of using resins doped with heteroatoms, providing LIG with new properties after laser induction.

Another mainstream of research concerns finding alternative precursors such as bioderived precursors for LIG. Promising results were obtained by converting lignin-based materials^51,95−98^ into LIG. The transition from synthetic to bioderived polymers could enable the use of inexpensive, edible,^43,99^ and transient^100,101^ sensors. Since the versatility of LIG lies in its possible modifications, the keystones of future development will be a systematic assessment of its synthetic and material procedures.

Now, research efforts are focused on producing LIG, refining it into useable sensors, and developing transduction methods and functionalization techniques that give adequate results and are reliable over time. As for every new sensor technology, this production technique still presents limitations in achieving a repeatable and consolidated process producing batches of LIG electrodes with the same properties. However, the use of commercially available lasers and precursors represents an advantage, compared to other sensor production techniques.
